# SLC7 transporters at the crossroads of amino acid metabolism and diabetes pathophysiology: insights and therapeutic perspectives

**DOI:** 10.3389/fnut.2025.1467057

**Published:** 2025-05-21

**Authors:** Tingting Xu, Xiaoshi Zhang, Qingqing Chen, Cheng Yang, Bo Deng, David G. Armstrong, Shunli Rui, Yueqin Zhou, Wuquan Deng

**Affiliations:** ^1^Department of Endocrinology and Metabolism, School of Medicine, Chongqing University Central Hospital, Chongqing Emergency Medical Centre, Chongqing University, Chongqing, China; ^2^Department of Surgery, Keck School of Medicine of University of Southern California, Los Angeles, CA, United States

**Keywords:** SLC7, diabetes mellitus, amino acid transporters, metabolism, mechanisms

## Abstract

Amino acids are fundamental components of all living cells, serving not only as the building blocks of proteins but also as crucial sources of energy and precursors to key metabolites and signaling molecules. Amino acid transporters, specialized membrane proteins, facilitate the movement of amino acids across plasma membranes and between various cells and organ compartments. The malfunction, absence, or overexpression of specific amino acid transporters is linked to several human diseases. Among the extensive family of solute carrier proteins (SLCs), which comprises 458 transporters, the SLC7 transporter family, inclusive of CATs (Cationic Amino Acid Transporters) and LATs (L-type Amino Acid Transporters), is particularly instrumental in cellular amino acid uptake. Disruptions in amino acid transport can lead to significant metabolic abnormalities in diabetes, characterized by impaired insulin signaling and altered glucose metabolism. A deeper understanding of amino acid transporters’ roles in metabolic processes and insulin signaling could shed light on the pathogenesis of diabetes and unveil novel therapeutic targets for this pervasive metabolic syndrome.

## 1 Introduction

The SLC7 transporter family, which includes several subfamilies such as the cationic amino acid transporters (CATs, SLC7A1–4) and the large neutral amino acid transporters (LATs, SLC7A5/8), plays a pivotal role in the cellular uptake of amino acids (1). These transporters are involved in a multitude of physiological processes, including protein synthesis, neurotransmitter production, and cellular signaling (2–4). Dysregulation of these transporters is increasingly recognized for its role in the pathogenesis of metabolic disorders such as diabetes.

Diabetes is marked by metabolic chaos, resulting in chronic hyperglycemia and compromised insulin signaling (5). This metabolic derangement is characterized by the defective handling of glucose, encompassing its uptake, utilization, and storage, as well as disturbances in both lipid and amino acid metabolism (6–8). These disruptions are central to the development of insulin resistance, β-cell dysfunction, and the hyperglycemic state that typifies diabetes mellitus (7, 9–11).

The role of amino acid transport is particularly critical in metabolic pathways and insulin signaling (12, 13). Amino acids not only provide the substrate for protein synthesis but also serve as precursors to various metabolic intermediates (14). Moreover, amino acids function as signal transducers, orchestrating metabolic pathways such as the mTOR pathway, a key regulator of cell growth and metabolic function (13, 15). Among the amino acid transporters, those belonging to the SLC7 family are of special importance due to their role in mediating cellular amino acid uptake, thereby directly affecting metabolic regulation and insulin signaling (16–18). The dysregulation of amino acid transport is implicated in the development of insulin resistance and, consequentially, diabetes (13, 19).

## 2 SLC7 transporters and metabolic function

### 2.1 Amino acid sensing and insulin signaling

Amino acid sensing is a critical cellular process involving the detection of amino acids through various signaling pathways, most notably by the mechanistic target of rapamycin complex 1 (mTORC1) (20–22). Amino acids activate mTORC1, a regulatory hub influencing protein synthesis and autophagy (23). Disturbances in amino acid sensing, particularly with branched-chain amino acids (BCAAs), have been linked to metabolic disorders, including insulin resistance and diabetes (22, 24–26).

Insulin signaling is another pivotal pathway, commencing with the binding of insulin to its receptor and governing glucose uptake, protein synthesis, lipid metabolism, and gene expression (27–29). Akt, an essential downstream effector of insulin signaling, is instrumental in facilitating glucose uptake into cells (28, 30–33). Dysregulation of this pathway is a contributing factor to insulin resistance and associated metabolic diseases (30, 34, 35), including Type 2 diabetes, non-alcoholic fatty liver disease (NAFLD), dyslipidemia, and obesity-related complications.

The interconnection between amino acid sensing and insulin signaling encompasses intricate interactions. BCAAs, for instance, affect insulin sensitivity and signaling, whereas insulin signaling modulates mTORC1 activity (36–38). The mTOR signaling pathway stands as a crucial mediator in the dialogue between insulin action and amino acid availability (20, 39, 40). Insulin activation of mTOR leads to the activation of ribosomal S6 kinase 1 (S6K1), promoting the phosphorylation of S6, which in turn regulates translation initiation (15, 41, 42). Notably, the activation of mTOR or S6K1 can lead to the phosphorylation of the insulin receptor substrate-1 (IRS-1) (43–45), thereby inhibiting insulin signaling pathways (46). Understanding the intricate crosstalk between these pathways is vital for elucidating the molecular basis of metabolic diseases and forging pathways toward targeted therapeutic interventions.

In this context, we highlight the significant role of the SLC7 transporter family in mediating cellular amino acid availability, thereby influencing insulin signaling and regulation of the mTOR pathway (20, 38, 40, 47–49) (Table 1).

**Table 1:** The specific functions of SLC7 transporters in metabolism-related pathway.

	Amino acid sensing	Insulin signaling	mTOR pathway (38, 49)
HATs (Lat1, Lat2)	Bind with 4F2hc (proper localization and function on the cell surface)	Increasing glucose uptake in response to insulin stimulation (promoting the transport of large neutral amino acids)	Considered to be major regulators of the amino acid-dependent mTORC1 signaling pathway
	Promote the trans-membrane transport of large neutral amino acids (Especially branched or aromatic amino acids)	Promoting the phosphorylation of insulin receptor subunits	Activating the mTORC1 signaling pathway by transporting amino acids (especially arginine and leucine)
			Promoting protein synthesis and cell proliferation
rBAT (SLC3A1)	Binding to LAT1 or LAT2 promotes the reabsorption of cystine by renal tubules (maintain the homeostasis of cystine levels)	The binding to LAT1 or LAT2 helps to enhance the efficiency of insulin signaling.	The presence of rBAT enhances the stability and function of LAT1 or LAT2 on the cell membrane, (enhanced the activation of the mTOR signaling pathway)
	rBAT ensures the proper functioning of HATs in the kidney (maintains the balance and metabolism of amino acids)		
4F2hc (SLC3A2)	4F2hc acts as a chaperone protein for LAT1 or LAT2, contributing to their proper folding, expression, and localization to the cell membrane.	The presence of 4F2hc helps protect LAT1 or LAT2 from protein degradation and enhances their stability on the cell membrane, thereby maintaining the normal function of the insulin signaling pathway.	Binding of 4F2hc helps protect LAT1 or LAT2 from protein degradation and enhances their stability on the cell membrane, (affecting the activation of the mTOR signaling pathway and protein synthesis)

### 2.2 Glucose homeostasis

Amino acids are known to potentiate insulin-mediated glucose uptake, particularly in the case of macroneutral amino acids such as leucine, valine, and isoleucine (50, 51). This effect is often attributed to the activation of the mTOR signaling pathway, which enhances the translocation of the glucose transporter GLUT4 to the cell membrane, thereby facilitating glucose uptake (15, 52–54). Additionally, a correlation has been observed, in several studies, between elevated amino acid levels-especially of branched-chain amino acids (BCAAs)-and the emergence of insulin resistance as well as metabolic syndrome (22, 26). A plausible explanation for this association is that excessive amino acid concentrations result in aberrant activation of the insulin signaling pathway, which, in turn, interferes with glucose uptake and metabolism (14, 17, 53, 55, 56).

Integral to the transport system are the large neutral amino acid transporters, LAT1, and LAT2, which share approximately 48% sequence homology (32, 57, 58). They form transporter complexes with 4F2hc, namely 4F2hc-LAT1 (SLC3A2-SLC7A5) and 4F2hc-LAT2 (SLC3A2-SLC7A8), which are essential for the proper localization of LATs to the plasma membrane (1, 3, 4, 59). LAT1 preferentially transports large neutral amino acids with branched or aromatic side chains, whereas smaller neutral amino acids are the substrates of choice for LAT2 (60–63). Fluctuations in amino acid concentrations can modulate various components of the insulin signaling cascade, such as the insulin receptor, insulin receptor substrate-1 (IRS-1), Akt, and mTORC1 (17, 33, 64). The presence of certain amino acids consequently enhances the functionality of the insulin signaling pathway, further promoting glucose uptake and metabolism (8, 9, 34).

In sum, current evidence indicates that shifts in amino acid metabolism can exert significant effects on glucose uptake and metabolism through a host of cellular mechanisms. For instance, studies have demonstrated that elevated levels of branched-chain amino acids (BCAAs) can disrupt insulin signaling pathways, thereby impairing glucose uptake in skeletal muscle cells (65, 66). This disruption may be associated with the activity of SLC7 transporters, such as LAT1 (SLC7A5), which facilitate the uptake of BCAAs into cells (67). When LAT1 is overactive, it can lead to an excessive influx of BCAAs, triggering metabolic stress and subsequent insulin resistance (68, 69).

Nevertheless, a more comprehensive understanding of the complex interactions among SLC7 transporter activity, amino acid concentrations, and glucose homeostasis remains a critical area for further investigation. Specifically, the precise regulatory mechanisms by which SLC7 transporters respond to dynamic changes in amino acid profiles and how these responses, in turn, modulate glucose metabolism across different tissues are not fully elucidated. Additionally, the role of post-translational modifications, such as phosphorylation and glycosylation, in fine-tuning SLC7 transporter function within the context of amino acid-glucose crosstalk requires in-depth exploration (70).

Such insights underscore the significance of maintaining amino acid equilibrium and transporter functionality in the management of metabolic health and disease. For example, in diabetes patients, dysregulation of SLC7 transporters and imbalances in amino acid metabolism may exacerbate hyperglycemia. Therefore, developing targeted therapies that can restore the proper activity of SLC7 transporters and rebalance amino acid levels could potentially offer novel strategies for improving glucose control and overall metabolic health.

## 3 SLC7 family members in diabetes pathophysiology

### 3.1 SLC7 transporter alterations in diabetes

Alterations in the expression or function of solute carrier family 7 (SLC7) transporters are implicated in the pathogenesis of diabetes, with effects spanning multiple tissues integral to glucose and amino acid metabolism (71, 72). Empirical evidence has confirmed the presence of impaired amino acid transport in the skeletal muscle of individuals with Type 1 Diabetes (T1D) (73–76). For example, studies have shown that specific mutations or dysregulation in SLC7 transporters can lead to reduced BCAA uptake in skeletal muscle cells, disrupting the normal amino acid-glucose metabolic crosstalk. Alterations in SLC7 transporter expression or activity within muscle tissues may contribute to the development of insulin resistance and broader metabolic dysfunction. In particular, the transporter LAT1 is critical for the uptake of branched-chain amino acids (BCAAs) like leucine, which play a significant role in insulin secretion from pancreatic beta cells. Elevated plasma levels of BCAAs, notably leucine, isoleucine, and valine, are commonly observed in individuals with Type 2 Diabetes (T2D) (16, 77–79). The increased BCAA levels are thought to activate certain intracellular signaling pathways, such as the mTOR pathway, which can lead to insulin resistance when overactivated (80). These increased amino acid levels correlate with insulin resistance and a heightened risk of developing T2D (81, 82). Additionally, Type 2 diabetes is often characterized by aberrant amino acid metabolism in the liver—a key site for regulating gluconeogenesis, lipid metabolism, and insulin sensitivity (83–85). Modifications in SLC7 transporter expression or functionality within hepatocytes exert profound influences on these metabolic processes. For instance, changes in the activity of SLC7A14, a member of the SLC7 family, can lead to the accumulation of lysosomal γ-aminobutyric acid (GABA), which impairs hepatic insulin sensitivity via inhibiting mTOR complex 2 (mTORC2)’s activity (86). It is also noteworthy that certain antidiabetic medications, such as metformin and thiazolidinediones, have been documented to affect amino acid metabolism and SLC7 transporter activity across various tissues (87–89). Metformin, for example, has been shown to restrict the tertiary control of BCAA cellular uptake by suppressing the activity of certain amino acid transporters, including those in the SLC7 family (90).

To sum up, the precise molecular underpinnings that link SLC7 transporter alterations to diabetes remain to be fully delineated. A deeper comprehension of these relationships holds the potential to unlock novel therapeutic targets and strategies for diabetes management.

### 3.2 SLC7 and insulin resistance

Numerous studies utilizing animal models and human biological samples have enhanced our understanding of the ways in which SLC7 transporter expression or function is altered in the context of insulin resistance. Skeletal muscle, a primary tissue responsible for insulin-stimulated glucose uptake, is a focal point of such research (91–93). Evidence suggests that in insulin-resistant animal models, the downregulation of SLC7A5 (LAT1) and SLC7A8 in skeletal muscle leads to impaired amino acid transport (94). This reduction in amino acid uptake can disrupt the activation of the mammalian target of rapamycin (mTOR) pathway, which relies on amino acid availability for its proper function (95). Since the mTOR pathway is closely intertwined with insulin signaling, its disruption can ultimately lead to decreased insulin sensitivity and impaired glucose metabolism (96–100).

**Figure 1:**
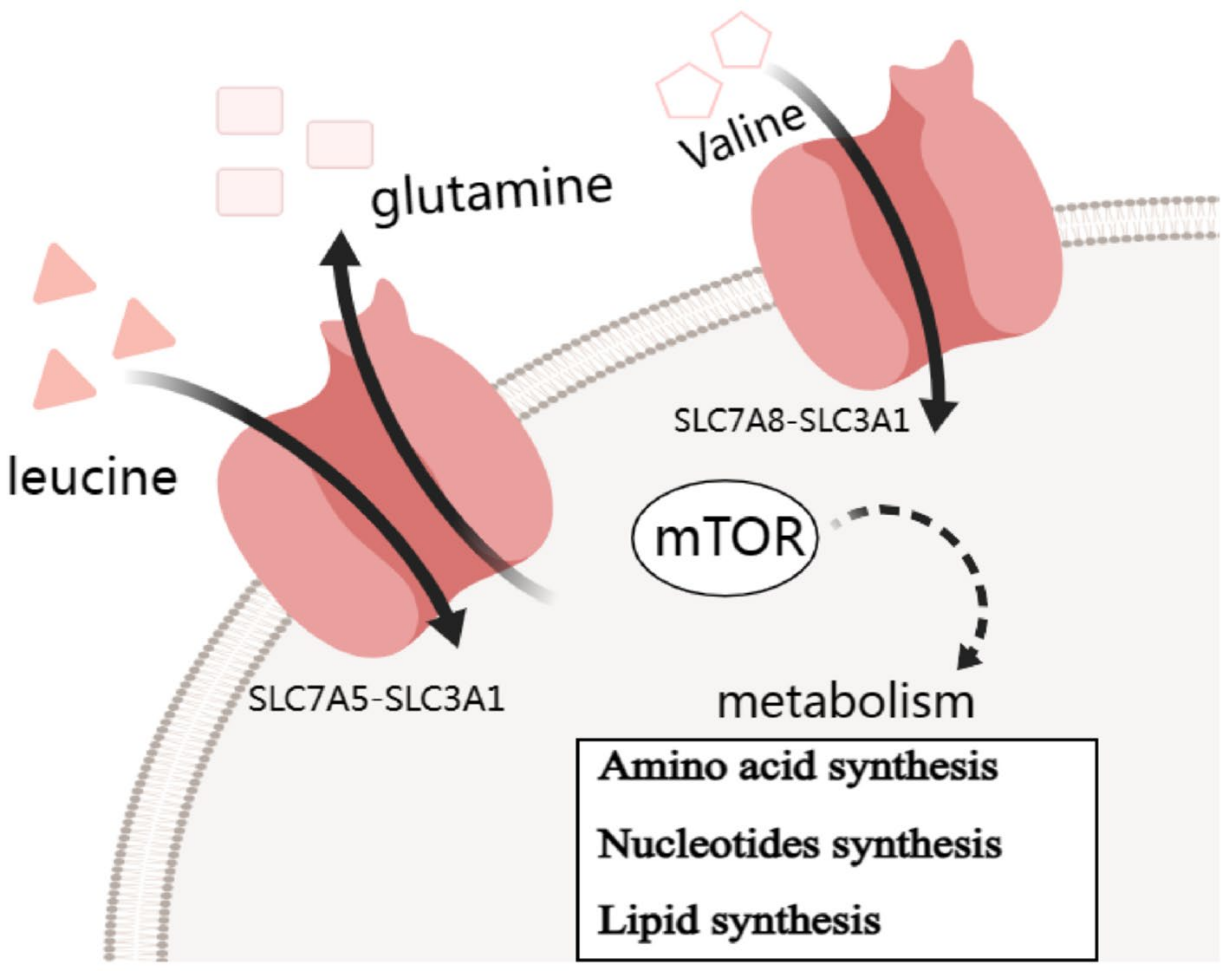
Simple carrier model for the mechanism of SLC7A5 and SLC7A8 transports changes contribute to insulin resistance by mTOR pathway.

Furthermore, research involving human adipose tissue has revealed changes in SLC7 transporter expression, notably of LAT1 (SLC7A5), in individuals affected by obesity and insulin resistance (70, 101, 102). LAT1-mediated amino acid uptake in adipocytes promotes the synthesis of triglycerides, contributing to adipocyte hypertrophy. Additionally, elevated amino acid levels taken up by LAT1 can trigger the activation of the nuclear factor kappa-light-chain-enhancer of activated B cells (NF-κB) pathway in adipocytes, leading to the production of pro-inflammatory cytokines (103). This chronic low-grade inflammation within adipose tissue is a key factor in the development of systemic insulin resistance (101, 104, 105) (Figure 1).

While LAT1 inhibitors present a promising therapeutic strategy, it is crucial to consider their potential side effects. For example, as LAT1 is involved in intestinal amino acid absorption, inhibition of LAT1 may disrupt this process, potentially leading to malnutrition (106). In addition, given the role of LAT1 in maintaining amino acid balance across the blood-brain barrier (107), its inhibition could affect the normal functioning of the central nervous system by altering the levels of amino acids available to the brain.

### 3.3 SLC7 and β-cell function

Amino acids, notably leucine and arginine, act as important secretagogues for insulin, inducing its release from pancreatic β-cells (108–111). Specific SLC7 family transporters, such as LAT1 (SLC7A5) and CAT-1 (SLC7A1), mediate the uptake of these amino acids into β-cells (110, 112–114). Once inside the β-cells, leucine, for example, binds to specific sensors, which then trigger a cascade of intracellular events. It activates the Sestrin2-GATOR2-GATOR1 axis, a key regulator in the mTORC1 activation pathway (115). This activation ultimately leads to the translocation of mTORC1 to the lysosomal surface, where it can interact with its upstream activator, Rheb-GTP (116). The SLC7-mediated amino acid transport is essential for this process, as a sufficient influx of amino acids is required to maintain the proper function of the sensors and downstream signaling components.

The activation of mTORC1 by amino acids, facilitated by SLC7 transporters, exerts multiple beneficial effects within β-cells. It stimulates protein synthesis through the phosphorylation of ribosomal protein S6 kinases (S6Ks) and eukaryotic initiation factor 4E-binding proteins (4E-BPs) (117–119). This enhanced protein synthesis is crucial for the production of various proteins involved in insulin biosynthesis and secretion machinery, such as insulin itself, proinsulin-converting enzymes, and components of the secretory granules. Moreover, mTORC1 activation also promotes β-cell growth and proliferation. It upregulates the expression of cyclins and cyclin-dependent kinases (CDKs), which are key regulators of the cell cycle progression (120). By enhancing β-cell mass and function, the SLC7-mTORC1 axis contributes to maintaining normal insulin secretion in response to physiological demands.

**Figure 2:**
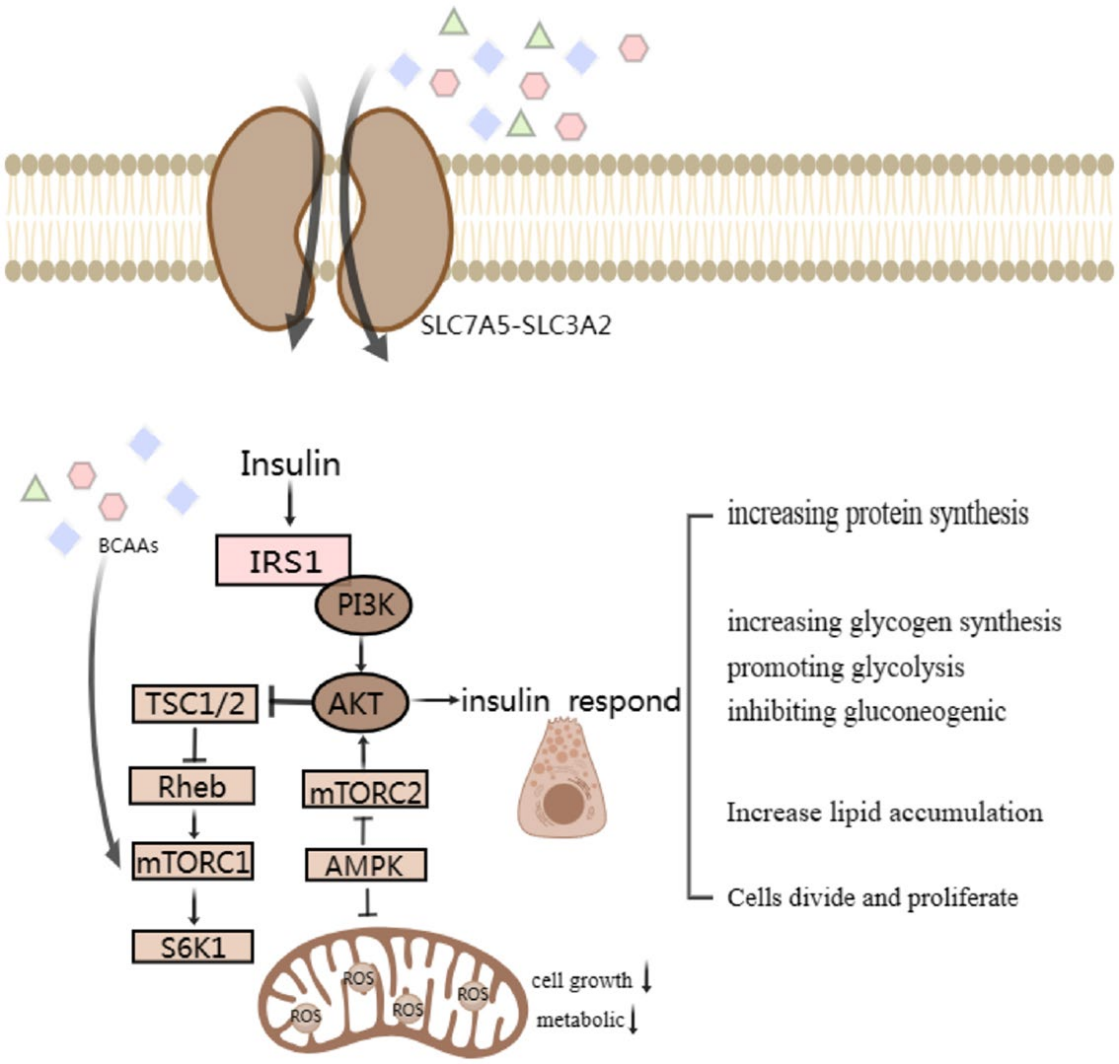
Simple signaling pathway map of SLC7 transporter protein changes affecting β-Cell Function and corresponding islet reactions. BCAA-activated mTORC1 and the following S6K1 phosphorylated insulin receptor substrate sites serine inhibit IRS-1. Modulating impaired protein kinase B, also known as Akt, activation through negative feedback attenuates insulin responses, such as increasing protein and glycogen synthesis, promoting glycolysis and lipid accumulation, affects cell differentiation and proliferation.

Beyond their role in amino acid transport, SLC7 transporters, such as SLC7A11 (also known as xCT), play a crucial role in the uptake of cystine, an essential precursor for the synthesis of the antioxidant glutathione (15, 121–124). Pancreatic β-cells are highly vulnerable to oxidative stress due to their relatively low antioxidant defense system and high endogenous production of reactive oxygen species (ROS) (125, 126). Oxidative stress can lead to DNA damage, protein oxidation, and lipid peroxidation within β-cells, ultimately resulting in impaired insulin secretion, β-cell dysfunction, and apoptosis. Cystine, once transported into the β-cells by SLC7A11, is reduced to cysteine. Cysteine is then incorporated into the glutathione synthesis pathway, where it combines with glutamate and glycine, catalyzed by specific enzymes, to form glutathione. Glutathione acts as a major intracellular antioxidant, scavenging ROS and maintaining the redox balance within β-cells (127–130). Thus, the transport of cystine via SLC7 transporters provides a vital protective mechanism against oxidative damage, safeguarding the normal function and survival of pancreatic β-cells (Figure 2).

## 4 Potential therapeutic implications

### 4.1 Drug targets now available inhibitors targeting

Inhibitors aimed at LAT1 are currently under consideration for cancer therapy, taking advantage of the transporter’s role in facilitating amino acid transport and supporting the proliferation of cancer cells (131–133). For instance, JPH203, developed in Japan, has entered clinical trials. It has shown potential in pre-clinical studies by blocking LAT1-mediated amino acid transport, thus impeding cancer cell growth and proliferation (131). Another example is the inhibitor developed by the research group at the University of Eastern Finland (134). This inhibitor was found to be hemocompatible and could induce apoptosis in cancer cells. It also has a more permanent structure and better selectivity for LAT1 compared to JPH203, as it does not undergo certain metabolic reactions that might weaken its anti-cancer effect. Additionally, compounds like 2-aminobicyclo-[2.2.1]-heptane-2-carboxylic acid (BCH) and α-(methylamino)-isobutyric acid (MeAIB) have been studied for their inhibitory effects on LAT1. BCH competitively inhibits LAT1’s transport function and has been used in research on cancer cell growth, while MeAIB can interfere with LAT1-mediated amino acid transport, although they may not be as potent as some other specific inhibitors (135, 136). Beyond oncology, these inhibitors may hold promise for treating metabolic disorders such as diabetes by influencing amino acid availability and modifying insulin signaling pathways (137). In addition to small molecule inhibitors, biologic agents, including antibodies and engineered proteins, present another avenue for targeting SLC7 transporters with specificity (71, 138, 139). These biologics could obstruct the transporter’s activity, tailor its substrate selectivity, or modulate associated signaling pathways. Particularly noteworthy is the potential of monoclonal antibodies directed against extracellular domains of SLC7 transporters to attenuate transporter function, thereby influencing metabolic processes governed by amino acid flux. A composite approach that combines therapies targeting SLC7 transporters with established treatments for diabetes—like insulin sensitizers and drugs that lower glucose levels—might produce compounded benefits. Such a strategy has the potential to offer a comprehensive treatment by tackling various dimensions of insulin resistance and metabolic dysregulation in diabetes patients.

### 4.2 Nutraceutical approaches

Dietary strategies that alter amino acid consumption may play a role in modulating SLC7 transporter activity, subsequently affecting metabolic health. Diets that are purposefully enriched with specific amino acids have been suggested to boost insulin sensitivity and enhance glucose regulation (19, 140, 141). The role of branched-chain amino acids (BCAAs) deserves particular attention, as high levels of circulating BCAAs are linked to insulin resistance and an increased risk of diabetes (17). Dietary adjustment of BCAA intake, or targeting their metabolic pathways, presents a compelling avenue for therapeutic interventions. There are many Dietary adjustment of BCAA intake, or targeting their metabolic pathways, presents a compelling avenue for therapeutic interventions. Emerging clinical evidence supports this notion. For instance, a study published in Diabetologia in 2023 demonstrated that metformin, a common anti-diabetic drug, suppresses the catabolic pathway of BCAAs in the liver of normal and obese mice, leading to BCAA accumulation and limiting its glucose-lowering efficacy. However, enhancing BCAA catabolism with small-molecule compounds or reducing BCAA intake through a low-BCAA diet significantly potentiated metformin’s anti-diabetic effects. Additionally, intermittent protein restriction, which reduces dietary BCAA intake, also notably improved the efficacy of metformin in treating Type 2 diabetes (77). Another recent study in 2025 from Gut Microbes, 30 participants at cardiometabolic risk were enrolled, and the MF diet group consumed cereal products rich in polyphenols, dietary fiber, slow-digestible starch, and ω-3 fatty acids. The results showed that the MF diet intervention significantly decreased serum BCAA levels, with leucine and isoleucine decreasing by 5 and 7%, respectively, (*p* < 0.05). This indicates that dietary modulation can effectively regulate BCAA levels, potentially improving insulin resistance and metabolic health (142). Furthermore, research on pancreatic ductal adenocarcinoma published in Nature Cell Biology revealed that targeting BCAT2, an enzyme involved in BCAA catabolism, or restricting BCAA intake in the diet could slow down the progression of the cancer in pre-clinical animal models. These findings not only highlight the role of BCAA metabolism in cancer development but also suggest that dietary and metabolic pathway-targeting strategies may have broader therapeutic applications beyond diabetes (143).

Interestingly, BCAAs are not the only components related to relevant transporters. Other reported components also play important roles. It has been reported that alliin, a component of garlic, is a novel substrate of SLC7A5 (144). This finding indicates that exploring a wider range of substrates of SLC7A5 could potentially open up new perspectives for therapeutic strategies, whether in diabetes treatment or other related fields. To sum up, adopting a personalized approach to nutrient intake that considers individual metabolic profiles and dietary preferences may improve the success rate of such interventions. Personalized nutritional recommendations designed to optimize amino acid consumption and modulate SLC7 transporter activity hold promise for superior metabolic outcomes, potentially leading to more effective diabetes management (145–148).

### 4.3 Diabetes treatment

In the realm of diabetes treatment, recent research has increasingly recognized the potential of targeting SLC7 transporters as a novel therapeutic strategy. For instance, emerging evidence suggests that small-molecule inhibitors designed to modulate the activity of specific SLC7 transporters, such as LAT1 (SLC7A5), can significantly improve insulin sensitivity and glucose homeostasis in pre-clinical diabetic models (80). These inhibitors work by altering the amino acid transport dynamics within key metabolic tissues. In skeletal muscle, by reducing the excessive influx of branched-chain amino acids (BCAAs) mediated by LAT1, they help alleviate the associated insulin resistance. A study in rodent models demonstrated that administration of a selective LAT1 inhibitor led to decreased plasma BCAA levels, enhanced insulin-stimulated glucose uptake in skeletal muscle, and ultimately, improved glycemic control (149). In the clinical context, understanding the role of SLC7 transporters also has implications for optimizing the use of existing antidiabetic medications. Genetic variations in SLC7 transporter genes have been shown to influence patients’ responses to drugs like metformin. Metformin, a first-line treatment for type 2 diabetes, has been reported to interact with SLC7 transporters in hepatocytes, modulating amino acid metabolism and contributing to its glucose-lowering effects (150). By delving deeper into these interactions, personalized treatment strategies can be developed to maximize the efficacy of antidiabetic drugs based on an individual’s genetic makeup and metabolic profile. Interestingly, while this review encompasses research on SLC7 transporters in both diabetes and cancer, there are notable overlaps in the molecular mechanisms regulated by these transporters in the two conditions. For example, the mTOR signaling pathway, which is crucial for β-cell growth and function in diabetes and is also involved in tumorigenesis, is influenced by SLC7-mediated amino acid transport in both contexts (59).

This shared pathway highlights the potential for cross-fertilization of research findings between diabetes and cancer fields. Insights gained from cancer research on SLC7 transporters, such as the development of targeted therapies, may offer new perspectives for diabetes treatment, and vice versa. This connection not only justifies the inclusion of cancer-related information but also enriches our understanding of the broader role of SLC7 transporters in human health and disease.

## 5 Research methods and models

### 5.1 Experimental techniques

Investigating the SLC7 family’s function and its influence on cellular metabolism, particularly amino acid transport, requires a diverse array of experimental methodologies. To assist researchers in gaining a deeper understanding of this significant biological family and to enrich the scientific toolkit with more robust data acquisition methods, we present a summary of experimental techniques applicable to the study of the SLC7 family. The foundational experimental approach involves transporter assays (151–153). These assays gauge the activity of SLC7 transporters by monitoring the uptake or efflux of specific amino acids. This category encompasses techniques such as uptake assays using radioactive or fluorescently labeled amino acids and electrophysiological methods designed to evaluate transporter kinetics and function. However, a critical consideration in such assays is the need to distinguish transporter-mediated uptake from intracellular metabolic processing (e.g., protein synthesis, catabolism). To address this, researchers often employ non-metabolic substrate analogues, such as α-methylated amino acids (e.g., α-methylleucine), which are recognized by transporters (e.g., LAT1) but cannot be incorporated into cellular metabolism (154, 155). For example, α-methylleucine specifically binds to LAT1 (SLC7A5) to measure transporter activity without interference from endogenous amino acid utilization (156, 157). When such analogues are not used, uptake data may reflect a combination of transport and metabolic fate, necessitating cautious interpretation of results. This category also encompasses electrophysiological methods designed to evaluate transporter kinetics and function, providing insights into ion-coupled transport mechanisms.

Moving toward metabolic analysis, metabolomic techniques offer a holistic assessment of metabolite profiles within cells or tissues, thereby allowing for an inference of SLC7 transporter impact on cellular metabolism (158–161). Mass spectrometry and nuclear magnetic resonance (NMR) spectroscopy are two prevalent tools used in these comprehensive metabolic studies (162–164).

Protein localization and trafficking studies provide critical information regarding the dynamics of SLC7 transporter distribution within cells in response to various stimuli (165, 166). Techniques such as immunofluorescence microscopy and subcellular fractionation assist in pinpointing the subcellular position of transporters (167, 168), Furthermore, genetic manipulation techniques, including gene knockout and overexpression, allow for direct control over SLC7 transporter expression in cellular or animal models. Pharmacological interventions utilizing inhibitors or activators can elucidate the functional significance of SLC7 transporters in cellular metabolism. Lastly, proteomic approaches reveal protein–protein interactions and post-translational modifications affecting SLC7 transporters, shedding light on their regulatory mechanisms (169–171).

### 5.2 Model systems

Animal models are indispensable tools for delineating the functions of the SLC7 family and offer invaluable perspectives on the transporters’ roles in metabolic disorders such as diabetes. Through an interdisciplinary approach that utilizes various model systems, researchers are able to gain precise insights into SLC7 transporter contributions to disease pathophysiology and target them for therapeutic interventions. Regarding *in vitro* studies, cell culture models, particularly insulin-responsive cell types like adipocytes and myocytes, are instrumental (172–174), Such cell lines are leveraged to understand glucose and amino acid metabolism regulation under varying expressions of specific SLC7 transporters. Manipulating SLC7 transporter expression in these cells—via overexpression or knockdown techniques—permits examination of their impact on both cellular metabolism and the insulin signaling cascade. Moreover, employing transporter and glucose uptake assays within these cell cultures enables analysis of the involvement of SLC7 transporters in the modulation of glucose and amino acid uptake. Turning to *in vivo* systems, animal models of diabetes, including mice with genetically induced type 1 or type 2 diabetes, deliver comprehensive insights into the systemic implications of SLC7 transporter dysfunction (175–178). Using genetic knockout or knock-in mouse models, researchers can expound on the specific SLC7 transporters’ influence on glucose equilibrium, insulin responsiveness, and β-cell physiology. Metabolic profiling in these models, including monitoring blood glucose, insulin, and amino acid concentrations, provides evidence of metabolic shifts linked to SLC7 transporter alterations (179–181). However, it is crucial to note the differences in SLC7 transporter expression patterns between rodents and humans. For instance, studies have shown that the expression of LAT1 (a member of the SLC7 family) in human islets is notably higher than that in mice (102). Additionally, the liver LAT2 activity in mice is significantly lower compared to humans (182), which may have a profound impact on the observed metabolic phenotypes in mouse models and limit the direct translation of findings to human conditions. These species-specific expression differences highlight the need for caution when extrapolating results from animal models to humans. Finally, human tissue samples, such as those from adipose tissue, skeletal muscle, and pancreatic islets, obtained from diabetic patients or healthy volunteers, present a direct window into SLC7 transporter activity in human metabolism. Transcriptomic and proteomic studies of these tissues aid in pinpointing variations in SLC7 transporter expression and functionality that are correlated with diabetes.

## 6 Challenges and future directions

### 6.1 Knowledge gaps

Despite the notable advancements in our grasp of how SLC7 family members influence the pathophysiology and management of diabetes, several significant knowledge gaps persist. Even though the expression of many SLC7 transporters is widespread, there is a notable lack of comprehensive understanding regarding their specific functions across various tissues and cell types. For instance, in the liver, while SLC7 family members are known to be involved in amino acid metabolism, the precise roles they play in regulating gluconeogenesis and lipid metabolism remain unclear. The interplay between SLC7 transporters and key hepatic signaling pathways under diabetic conditions, such as the PI3K-AKT and AMPK pathways, has not been fully elucidated. In skeletal muscle, a major site of insulin-mediated glucose uptake, the contribution of different SLC7 transporters to the regulation of amino acid-induced insulin sensitivity and glucose disposal is poorly understood. It remains uncertain how alterations in SLC7 expression and function impact muscle protein synthesis and breakdown, which are critical processes in the context of diabetes-associated muscle wasting. Regarding islet beta cells, which are essential for maintaining glucose homeostasis through insulin secretion, the specific functions of SLC7 family members in beta cell development, proliferation, and survival are still largely unknown. Whether SLC7 transporters directly modulate insulin granule biogenesis and exocytosis, and how their dysregulation contributes to beta cell dysfunction and failure in diabetes, are questions that have yet to be thoroughly explored.

Additionally, the intricacies of how SLC7 transporter expression and activity are modulated by metabolic stimuli, hormonal influences, and during pathological states, still elude us. A deeper dive into the molecular mechanisms governing the regulation of SLC7 transporters stands to uncover critical insights into their involvement in diabetes pathogenesis. Such discoveries may pave the way to identifying new therapeutic targets and refining treatment options.

### 6.2 Emerging research

The latest insights from research focused on SLC7 transporters are shedding meaningful light on their involvement in metabolic disorders, including obesity, diabetes, and metabolic syndrome, providing promising directions for clinical intervention. The burgeoning evidence underscores the significance of these transporters in disease etiology, thus warranting investigations into the precise mechanisms by which SLC7 transporter dysregulation precipitates metabolic imbalance. Upcoming studies should aim to disentangle their roles in nutrient detection, energy homeostasis, and the intricate web of insulin signaling pathways. Research has only recently started unmasking the sophisticated regulatory factors that dictate SLC7 transporter function. Notably, this includes exploring the realm of post-translational modifications, protein–protein binding events, and transcriptional governance. Further research endeavors should drill deeper into these areas to map out detailed regulatory landscapes. The goal will be to identify viable molecular targets through which SLC7 transporter activity can be adjusted to better manage and potentially treat metabolic conditions.

## 7 Conclusion

The SLC7 family of transporters plays a critical and multifaceted role in metabolic processes that are highly relevant to diabetes, primarily through mechanisms linking amino acid sensing, insulin signaling, and pancreatic β-cell function. These transporters, such as LAT1 (SLC7A5) and CAT-1 (SLC7A1), mediate the uptake of essential amino acids (e.g., leucine, arginine) that activate the mTORC1 signaling pathway—a central hub for integrating nutrient availability with insulin secretion and glucose metabolism. For example, leucine transported by LAT1 in pancreatic β-cells promotes insulin granule biogenesis and β-cell proliferation, while in skeletal muscle, SLC7-mediated branched-chain amino acid uptake maintains mTORC1-dependent insulin sensitivity, preventing downstream insulin resistance via IRS-1 serine phosphorylation. Additionally, the cystine transporter xCT (SLC7A11) safeguards β-cells from oxidative stress by supporting glutathione synthesis, a critical antioxidant defense against glucolipotoxicity-induced apoptosis.

Beyond these mechanisms, SLC7 transporters regulate broader metabolic processes, including glucose homeostasis, inflammation, and lipid metabolism. Members of the family are responsible for transporting branched-chain amino acids (leucine, isoleucine, valine), whose dysregulated levels correlate with insulin resistance and β-cell dysfunction in type 2 diabetes. Their expression in key metabolic tissues—such as pancreatic β-cells, skeletal muscle, and adipose tissue—highlights their role in maintaining metabolic equilibrium, from modulating insulin secretion to influencing glucose uptake and storage.

SLC7 transporters also extend their functions beyond metabolism, impacting neurotransmitter transport, synaptic efficacy, and brain amino acid balance, positioning them as promising targets for neurological disorders like epilepsy and Alzheimer’s disease. However, this review acknowledges limitations: while preclinical studies underscore their therapeutic potential, clinical trials remain scarce, and research has disproportionately focused on cancer rather than endocrine and metabolic diseases. Future investigations should prioritize clinical research and mechanistic studies in diabetes and related disorders, bridging the gap between foundational discoveries and translational applications. By elucidating the precise roles of SLC7 transporters in amino acid–glucose crosstalk and β-cell survival, we can unlock novel strategies to restore metabolic health and address unmet therapeutic needs in diabetes and beyond.
